# Preexposure to one social threat alters responses to another social threat: Behavioral and electrophysiological evidence

**DOI:** 10.3758/s13415-023-01151-y

**Published:** 2024-01-10

**Authors:** Xu Fang, Rudolf Kerschreiter, Yu-Fang Yang, Michael Niedeggen

**Affiliations:** 1 Department of Education and Psychology, Division of Experimental Psychology and Neuropsychology, Freie Universität Berlin, Habelschwerdter Allee 45, 14159 Berlin, Germany; 2 Department of Education and Psychology, Division of Social, Organizational, and Economic Psychology, Freie Universität Berlin, Berlin, Germany

**Keywords:** Cyberball, Loss of control, Social exclusion, Preexposure, Expectancy violation

## Abstract

A recent Cyberball study has indicated that the experience of loss of control can affect how people process subsequent social exclusion. This “preexposure effect” supports the idea of a common cognitive system involved in the processing of different types of social threats. To test the validity of this assumption in the current study, we reversed the sequence of the preexposure setup. We measured the effects of social exclusion on the subsequent processing of loss of control utilizing event-related brain potentials (ERPs) and self-reports. In the control group (CG, *n* = 26), the transition to loss of control elicited significant increases in both the P3 amplitude and the self-reported negative mood. Replicating the results of the previous preexposure study, these effects were significantly reduced by the preexposure to an independent social threat (here: social exclusion). In contrast to previous findings, these effects were not modulated by the discontinuation (EG1_disc_, *n* = 25) or continuation (EG2_cont_, *n* = 24) of the preexposure threat. Given that the P3 effect is related to the violation of subjective expectations, these results support the notion that preexposure to a specific social threat has widespread effects on the individuals’ expectancy of upcoming social participation and control.

**Supplementary Information:**

The online version contains supplementary material available at 10.3758/s13415-023-01151-y.

Social interactions often involve coping with various threats, including social exclusion and loss of control. The former refers to the experience of being excluded or ignored by individuals or social groups (Williams, 2009); the latter refers to the inability to choose a preferred option or being overruled (Inesi et al., 2011; Rotter, 1966). These threats can jeopardize fundamental human needs, such as “belonging,” “self-esteem,” “control,” and “meaningful existence.” Consequently, these threats can lead to negative arousal and affect both physical and mental health (Baumeister & Leary, 1995; DeWall et al., 2011; Leotti et al., 2010; Struk et al., 2021; Williams et al., 2000).

Several theories have been proposed to account for the aversive effects driven by social threats. According to the seminal model of Williams (2009), social exclusion elicits a reflexive response based on the activation of a preattentive alarm system. This account has been supported by brain imaging (Eisenberger, 2012) and ERP approaches (Themanson et al., 2013). Other—nonexclusive—accounts suggest that the aversive response is based on an inconsistency between the expected and experienced participation (Kerr & Levine, 2008). This approach, which will be detailed below, also is embraced by recent common expectancy violation models (Panitz et al., 2021; Proulx et al., 2012).

Neither account provides clear predictions about whether different types of threat, such as exclusion or loss of control, are processed separately or whether a common system is involved. Following a neuroimaging approach (Oliveira et al., 2007), the similarity of brain activation patterns driven by different threats could be explored. Our approach utilizes the concept of “preexposure” to probe the integration within the cognitive system, a technique that builds upon the concept of “social priming” (Graham & McLaren, 1998; Weingarten et al., 2016). Priming implies that the processing of a “target” event can be facilitated (e.g., semantic priming, Ferrand & New, 2003) or impeded (e.g., negative priming, Mayr & Buchner, 2007) by the presentation of a preceding stimulus—related to the target stimulus. In our case, the preexposure effect refers to the pre-experience of participants before entering a social aversive situation. Please note that we used the term “preexposure” instead of “priming,” which is more closely related to the preactivation of semantic and/or perceptual representation (Farah, 1989; McRae & Boisvert, 1998).

In a recent preexposure study, Fang et al. (2022) used behavioral and electrophysiological methods to identify preexposure effects of loss of control on the processing of social exclusion. Notably, both threats (loss of control and exclusion) were triggered by using a common experimental setup based on the Cyberball paradigm. In the original version (“exclusionary Cyberball”) of this virtual ball-tossing game established by Williams and Jarvis (2006), participants engage in an online game with two putative co-players who are actually computer-generated. By reducing the frequency of participants’ ball receptions, a sense of exclusion is induced, primarily threatening the need to “belonging” that is typically identified with the psychological experience of gaining acceptance and avoiding rejection (DeWall et al., 2011; Hartgerink et al., 2015; Wesselmann et al., 2023). More recently, a modified Cyberball setup (“intervention Cyberball”) introduced a “supervisor” who could override the participant’s decision and choose a different recipient for the ball toss (Niedeggen et al., 2019). This manipulation generates a sense of diminished autonomy, thereby threatening the need for “control.” The threats to basic needs (“belonging” or “control”) are measured by a standardized questionnaire: the Need-Threat Questionnaire (NTQ) (Williams, 2009). The compatibility of both setups enables researchers to investigate social exclusion and loss of control in a single paradigm, facilitating the examination of interaction effects between them.

This idea was first tested in the recent preexposure study (Fang et al., 2022), which was based on the idea that the processing of a specific social threat would affect the processing of an upcoming different threat if both are represented in a common cognitive system. This notion is related to a process of “social priming” or preexposure. In other words, participants who first experienced “intervention Cyberball” displayed a modulated response to the following “exclusionary Cyberball,” attesting to the influence of previous social threats on the perception of a novel social threat. The experimental conditions are presented in Fig. 1 (see below). The results indicated that the processing of social exclusion was influenced by the preceding experience of loss of control. In particular, the self-reported threat to “belonging” and negative mood were significantly reduced when the exclusion was preceded by preexposure to loss of control. Interestingly, the preexposure effect was not restricted to the self-reports but was expressed in event-related brain potentials (ERPs), which are closely linked to the processing of exclusionary events.

**Fig. 1:**
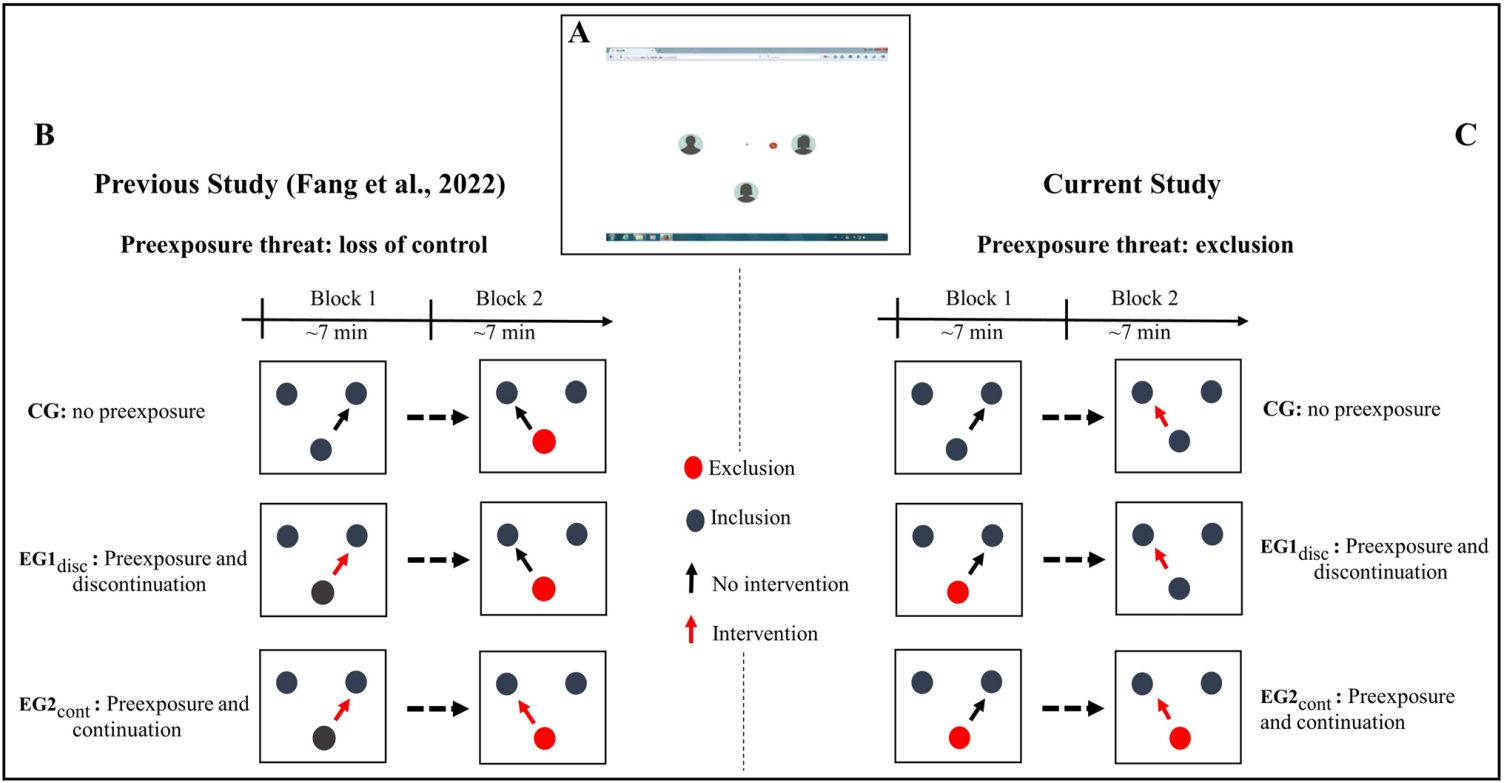
Comparison of the experimental setup of the previous and current preexposure study: (**A**) Experimental setting. The display imitated an Internet screen with a ball icon and three avatars. The avatars of two putatively connected co-players were vertically centered and the participant’s avatar was presented at the bottom of the screen and in a horizontally centered position. The symbol of the ball in spatial proximity to one avatar signaled the possession of the ball. (**B**) Experimental design in the previous study (Fang et al., 2022). Preexposure in block 1 (EG1_disc_ and EG2_cont_) was defined by the intervention (indicated by the red arrow). In block 2, participants in all groups received a partial exclusion threat (indicated by the red dot). To control the effect of the offset of the preexposure threat, the loss of control continued in EG2_cont_. (**C**) Experimental design in the current study. In block 1, no intervention occurred (indicated by the black arrow), but participants assigned to experimental groups (EG1_disc_ and EG2_cont_) were partially excluded (indicated by the red dot). In block 2, participants in all groups received a partial loss-of-control threat (indicated by the red arrow). Again, to control the effect of offset of the preexposure threat, exclusion continued in EG2_cont_. *Notes.* CG: control group without preexposure; EG1_disc_: experimental group 1 with discontinued preexposure; EG2_cont_: experimental group 2 with continued first threat

In line with previous studies (Kawamoto et al., 2013; Themanson et al., 2013; Weschke & Niedeggen, 2015), the ERP component most sensitive to the preexposure state is the P3. The P3 “complex” is characterized by the number of frontal and centroparietal components. The more-frontal components (P3a) occur at approximately 300 ms and have been linked to the early activation of the attentional mechanism (Friedman et al., 2001). The posterior components (P3b) follow the P3 deflection and have been related to updating, mnestic processing, or closure (Donchin & Coles, 1988; Verleger, 2020). Previous studies suggest that both P3 components are elicited in the Cyberball paradigm: A frontal P3 occurring between 240 and 300 ms (Gutz et al., 2011; Jenkins & Obhi, 2020; Weschke & Niedeggen, 2013) was followed by a centroparietal P3 in the time range 300–400 ms (Gutz et al., 2011; Kawamoto et al., 2013; Weschke & Niedeggen, 2013). More importantly, their amplitudes are related to the subjective probability of relevant events (Gutz et al., 2011; Themanson et al., 2013).

Specifically, P3 amplitudes increase when participants receive the ball less frequently, as seen in the “exclusionary Cyberball” (Schuck et al., 2018), or when participants experience more frequent interventions in their intended ball throwing, as observed in the “intervention Cyberball” (Niedeggen et al., 2019). Importantly, the increase in P3 amplitudes (hereafter referred to as the P3 effect) induced by the transition to an exclusion and/or intervention condition is not a mere reflection of a change in the perceived probability of the event but reflects the violation of subjective beliefs and expectations: The P3 effect is not observed if the actual situation meets the participants’ expectations, for instance, when a reduced probability of ball reception can be attributed to an increase in the number of co-players (Weschke & Niedeggen, 2015). Conversely, the P3 effect can be enhanced if the participants’ expectancy levels are elevated, such as by inducing a sense of entitlement through a superior position (Niedeggen et al., 2017, 2019). In sum, the effects are congruent with the idea that P3 components allow us to track changes in the level of individual expectancies (Mars et al., 2008).

In the aforementioned preexposure study (Fang et al., 2022), ERP results support the idea that a common system is involved in the processing of both social threats. As mentioned, the self-reports already showed that the preexposure to loss of control affected the processing of upcoming exclusionary signals: the preexposure reduced the self-reported threat to “belonging.” In line with the self-reports, the P3 effects evoked by ball receptions in an exclusionary condition were significantly reduced. This is congruent with the idea of the “Violation-of-Expectation Model” (ViolEx, Panitz et al., 2021), in which situation-specific expectations trigger internal anticipatory reactions.

The previous study (Fang et al., 2022) also signaled that the expression of the preexposure effect depends on the “fate” of the first threat: As shown in Fig. 1, the preexposure either stopped with the onset of the second threat (discontinued threat) or overlapped with the second threat (continued threat). The latter (continued threat) appears to confirm the previously built expectation on social participation and control, and the reduction of the above-mentioned P3 effect induced by a transition to social exclusion was clearly expressed. However, in the case of an offset of the preexposure threat (discontinued threat), the reduction of the P3 effect was less expressed. The authors supposed that the discontinuation of the preexposure threat questions the previously established expectation and leads to a restoration of the “default” expectancy state. This modulation of the preexposure effect also can be explained using the theoretical framework of the ViolEx model (Panitz et al., 2021): the offset of the preexposure threat represents a pleasant surprise (Garrett & Sharot, 2017), which weakens the validity of the previously established internal negative expectation and increases sensitivity to the new threat. Notably, the reduced P3 effects do not correspond to the reduced effects in self-reports; on the contrary, the temporal overlap of the two threats significantly increases the reported threat to “belonging” and negative mood.

The current research tries to replicate and extend previous research on the preexposure effect. If the assumption of a common expectancy system is correct, similar psychophysiological and self-reported effects should be observed when the order of presentation of the two threats is reversed, such that the experience of social exclusion precedes the processing of a subsequent loss of control.

Moreover, reversing the order provides an opportunity to test more directly whether different social threats—serving as preexposure stimuli—have different strengths with respect to the effects (self-reports, ERPs) elicited. In other words, the experimental setup allows a comparison of the valence of the two threats. Previous research indicates that social exclusion is a potent threat that might affect mental health (Ademiluyi et al., 2022; MacDonald & Leary, 2005) and corresponding effects of a sustained “loss of control” might be related to the concept of “learned helplessness” (Baratta et al., 2023). Previous Cyberball studies rather suggest differences in valence of these threats: In contrast to interventions that specifically challenge the need for “control” (Niedeggen et al., 2019), the effects of social exclusion spread to a wide range of social needs (Niedeggen et al., 2023; Weschke & Niedeggen, 2013; Williams, 2007, 2009), including “belonging.” “self-esteem,” and also “control.” Consequently, preexposure to exclusion might have a more severe impact on expectancy levels compared with preexposure to loss of control.

Based on these premises, we predict that the experimental effects of preexposure observed in the previous study (Fang et al., 2022) can be replicated if the order of the two threats is reversed. These effects can be attributed to an adaptation effect in a common expectancy system. Furthermore, we predict that the expression of the preexposure effect will depend on the continuation or discontinuation of the preexposure threat. Accordingly, the two following hypotheses were formulated:

- ***Hypothesis 1:**** Preexposure to social exclusion will reduce the P3 effect elicited by the transition to loss of control. This reduction in the P3 effect will be accompanied by reduced effects on self-reported threat to “control” and negative mood.* In other words, the P3 effects, the threat to “control” and negative mood will be expressed more strongly in the control group than in the experimental groups, because without preexposure, the control group is not prepared for the upcoming social threat.
- ***Hypothesis 2:**** Continuation of the preexposure threat (exclusion) will lead to pronounced reductions of the P3 effect as well as of self-reported threats in response to the new threat (loss of control). In contrast, if the onset of the new threat (loss of control) is associated with the offset of the preexposure threat (exclusion), the aforementioned effects (reductions in P3 effects and self-reported measures) will be less expressed.*

Moreover, we will conduct an exploratory analysis to compare the expression of ERP effects and self-reported effects elicited by preexposure to exclusionary signals with the previously reported effects of preexposure to interventional signals (Fang et al., 2022). This analysis will provide preliminary evidence on whether exclusionary signals have a greater impact on social expectations compared with interventional signals.

## Methods

We obtained approval for the research protocol from the local ethics committee of the Freie Universität Berlin (No.006.2019, May 15, 2019). Following the tenets of the Declaration of Helsinki, all participants gave written, informed consent before and after the experiment. The dataset, including self-reports, preprocessed EEG data, program, and analysis codes, is available at https://osf.io/dbmcr/ (date of access: August 21, 2023).

### Participants

Sample size calculations were conducted a priori by using the G*power software (Erdfelder et al., 1996). The effect size estimation was based on a previous study (Fang et al., 2022) employing a 3 × 2 between-within design. In line with the previous study, a minimum of 66 participants was required to achieve 80% power at an alpha level of .05, assuming a medium effect size (*f* = 0.20, adjusted to Cohen’s taxonomy) for the anticipated interaction. Anticipating a rejection of approximately 20% of the data sets following a strict EEG artifact removal criteria (Niedeggen et al., 2023; Weschke & Niedeggen, 2016), a total sample of 95 participants (65 females, 30 males; age range: 18–56 years; *M*_age_ = 24.65 years, *SD*_age_ = 5.55 years; all right-handed except 10 participants) was recruited. All participants were randomly assigned to the three groups (control group without preexposure: CG; experimental group 1 with discontinued preexposure: EG1_disc_; experimental group 2 with continued first threat: EG2_cont_). They were rewarded with either credit points or cash (€ 20) for completing the entire task.

All participants were undergraduate students studying at universities in the Berlin area aged 18 to 40 years. In the current study, as well as in the previous study (Fang et al., 2022), we considered the potential impact of age on the neural characteristics revealed in the ERPs (Rodrigo et al., 2014; Vijayakumar et al., 2017). Therefore, we recruited participants in a predefined age range (18–40 years) to minimize this source of variability. Participants were fluent in German, English, or Chinese with normal or corrected-to-normal visual acuity and without concurrent acute psychological disorders or medical conditions that could potentially confound the study results. Because the participants (95 in total) had diverse mother tongues (German: 53, English: 22, Chinese: 20), the language of instruction was adapted accordingly. Post hoc inspection confirmed that participants with different mother tongues were almost equally distributed across three groups. (see Table S1 in the *Supplementary Material*).

One participant was excluded from the analysis because of exceeding the age criterion (>40 years). Additionally, 19 participants (12 females, 7 males; age range 18–32 years; *M*_age_ = 24.95 years, *SD*_age_ = 4.48 years) had to be rejected after a rigorous EEG artifact correction (criteria: see below). The final sample consisted of 75 participants (52 females, 23 males; age range 18–40 years; *M*_age_ = 24.16 years, *SD*_age_ = 4.56 years; all right-handed except 8 participants), providing greater statistical power, as the rejection rate was lower than expected. The distribution across groups is as follows: 26 participants (14 females) in CG, 24 participants (17 females) in EG1_disc_, and 25 participants (21 females) in EG2_cont_.

### Procedures

The experimental procedure was developed using PsychoPy2 (version V 1.85.6, Peirce, 2007), and the Cyberball setup was adapted for EEG measurements based on previous studies: First, it considered a reduction in ball reception (partial exclusion) instead of total exclusion (no ball reception) (Gutz et al., 2011). Second, it embedded loss of control (intervention Cyberball, Niedeggen et al., 2019). Note that a similar experimental design with a different preexposure (loss of control) has already been established in a previous study (Fang et al., 2022).

The setup of the Cyberball game is illustrated in Fig. 1A. The participant was represented by an avatar (a sketch of a human head), which was always presented in the lower part and horizontally centered position on the computer screen. Participants were connected via the Internet to two putative co-players whose avatars were vertically centered. The spatial distance between the three avatars remained constant, and the viewing angle was fixed at 3°, restricting eye movements within this range. To simulate the ball-throwing game, a corresponding ball symbol was displayed, and its spatial proximity to an avatar indicated ball possession. In the case of the participant’s ball possession, they could pass the ball to one of the two co-players by pressing the left or right arrow key on the keyboard (the “left arrow” indicated the intention to pass the ball to the left co-player, while the “right arrow” indicated the intention to throw the ball to the right co-player). After pressing the arrow key, the ball first disappeared for 500 ms and then reappeared next to one co-player. To enhance the validity of the setup, the putative co-players held the ball for a random duration between 400 ms and 1,400 ms.

All participants were given a cover story. They were informed that they were taking part in a study of visual imagery abilities and were asked to complete a questionnaire (Visual Imagery Vividness Questionnaire; Marks, 1973). Following the instructions, participants were fitted with an EEG cap, and electrodes were attached. They were then seated in front of a computer monitor (visual angle 7° × 7°, viewing distance of 120 cm) with their chin resting on a height-adjustable chin rest. To foster participants’ sense of “control” during the game, they were instructed to select one of six avatars to represent themselves in the subsequent game (Lim & Reeves, 2009). In addition, all participants were informed that an independent “supervisor”—who was not one of the co-players—was involved in the game. The “supervisor” could interfere with their decisions, and this intervention would result in the ball reception of the nonintended co-player. This manipulation was intended to lead to a selective threat of the need for “control” (Niedeggen et al., 2019). The role of the “supervisor,” which is actually controlled by the computer program, is personified in the instruction to solidify the cover story that includes virtual co-players. Moreover, this instruction has already been established in previous intervention Cyberball (Fang et al., 2022; Niedeggen et al., 2019; 2023). Its continued use supports a comparison of the results of the current study with previous preexposure studies (Fang et al., 2022). Additionally, the analysis of the first preexposure study (Fang et al., 2022) ruled out that exclusion is attributed to the activity of the “supervisor.” Even if the “supervisor” is “active,” exclusionary signals elicit a significant threat to “belonging.”

To ensure familiarity with the rules of the game and to maintain comparable expectations on participation and control, participants first completed a short practice session (100 throws in total). The practice block was set to an inclusive condition (i.e., 33% ball reception for each of the 3 players, 33 times each), and no intervention occurred (i.e., 0% intervention). The following two experimental blocks (Fig. 1C) consisted of 200 trials each. To support the cover story, each experimental block was preceded by a visual picture of the scene (grass or beach, presented in counterbalanced order) and corresponding written instructions (i.e., “Imaging to play the ball-tossing game on the beach”).

In the first experimental block (block 1), participants in CG remained in the inclusion condition (33% ball reception per player, i.e., each player received the ball approximately 66 times), whereas participants in both EG1_disc_ and EG2_cont_ experienced a partial exclusion condition (17% ball reception for the participant, i.e., participants received the ball only approximately 34 times). All participants in the three groups did not experience any interference in this block, i.e., the ball always reached the intended co-player. In the second experimental block (block 2), all participants—independently of the group assignment—experienced partial loss of control (probability of interference: 30%). In addition, the participants assigned to CG remained in an inclusionary condition, i.e., the ball reception rate was also 33% in block 2 (66 times of ball reception; 30% interference resulted in a total number of 46 intended receipts and 20 nonintended receipts). Participants in EG1_disc_ were transitioned to an inclusionary condition, i.e., the participants’ possession rate increased from 17% in block 1 to 33% in block 2 (66 times of ball reception in block 2; 30% interference resulted in a total number of 46 intended receipts and 20 nonintended receipts). Participants in EG2_cont_ continued to experience exclusion in block 2, with the probability of ball reception remaining at 17% (34 times of ball reception; 30% interference resulted in a total of 24 intended receipts and 10 nonintended receipts).

Immediately following block 2, participants completed a series of retrospective questionnaires presented on the screen. The first questionnaire was the Needs-Threats-Questionnaire (NTQ, (Hartgerink et al., 2015; Williams et al., 2000)), which measures threats to “belonging” (3 items) and “control” (3 items), respectively. Other individual scales included in the NTQ (“self-esteem” and “meaningful existence”) were left out, because no specific hypothesis was related to these corresponding threats. Next, the Self-Assigned Personal Power Questionnaire was administered (2 items, e.g., “I felt independent”; Lammers et al., 2009). The third one was the Negative Emotions Scale (8 items, e.g., “I felt sad” or “I felt angry”; Watson et al., 1988). For all self-report scales, participants were asked to estimate changes in feelings between the blocks on a 7-point Likert scale (ranging from −3 “stronger in block 1” to 3 “stronger in block 2”). This relative judgment has been established in previous studies (Fang et al., 2022; Niedeggen et al., 2023). In addition, participants had to estimate the frequency of ball reception and intervention in both blocks (manipulation check).

Please note that participants with different first languages were recruited (see above). To account for this difference, the self-reports were administered in the corresponding language (German, English, or Chinese). The German (Rudert & Greifeneder, 2016) and Chinese (Xu et al., 2017) adaptations of the original NTQ scales (Williams, 2009) were employed to measure these self-reports. Statistical analyses indicated that all self-reports in this study were not moderated by the factor of “language” (“control”: *F*(4, 66) = 0.316, *p* = 0.866, *η*_p_^2^ = 0.019 (90% confidence interval (CI) [0, 0.034]), “belonging”: *F*(4, 66) = 0.542, *p* = 0.705, *η*_p_^2^ = 0.032 (90% CI [0, 0.068]), “negative mood”: *F*(4, 66) = 0.776, *p* = 0.545, *η*_p_^2^ = 0.045 (90% CI [0, 0.094]), and “personal power”: *F*(4, 66) = 0.450, *p* = 0.772, *η*_p_^2^ = 0.027 (90% CI [0, 0.055])).

### EEG recording and data preprocessing

The setting and procedures of analysis were identical to the previous preexposure study (Fang et al., 2022) to allow for a comparison of results. Ag/AgCl electrodes were attached to an elastic electrode cap (EASYCAP, Herrsching, Germany) and connected to a digital amplifier (BrainAmps amplifier, Brain Products, Gilching, Germany). EEG signals were recorded from eight active electrode sites (AFz, Fz, F3, F4, Cz, Pz, P7, P8) (Fang et al., 2022; Niedeggen et al., 2023). The EEG signals obtained from the active electrodes were referenced to the linked earlobes, with FCz serving as the ground electrode (Niedeggen et al., 2019; Niedermeyer & da Silva, 2005; Schuck et al., 2018). To enhance the quality of the EEG signal and to reduce noise, impedances of all electrodes were kept below 10 kΩ (Gutz et al., 2011). The recording setup included vertical and horizontal electrooculograms (EOGs) to monitor for ocular artifacts, such as blinks and eye movements (Themanson et al., 2013; Weinbrecht et al., 2018). The EEG data were continuously sampled at a rate of 500 Hz and online filtering was performed by using a 0.1–100-Hz bandpass filter and a 50-Hz notch filter to reduce the effect of AC hum (Niedeggen et al., 2023; van den Berg et al., 2014).

Offline EEG analysis was performed by using Brain Vision Analyzer software (version: 2.1, Brain Products, Gilching, Germany). The EEG data in two experimental blocks were filtered through a bandpass filter with a cutoff range of 0.3 to 30 Hz and 24 dB/oct. This filter setting is congruent with earlier studies on the P3 component (Caesarendra, 2017; Kiat et al., 2017; Mars et al., 2008; Niedeggen et al., 2023; Weinbrecht et al., 2018; Weschke & Niedeggen, 2015) and—more importantly—are essential to allow a comparison with the data of the previous preexposure study (Fang et al., 2022). For EEG data in block 2, epochs (−100 to 800 ms) were created based on the onset of two events: “ball reception by the intended co-player” and “ball reception by the non-intended co-player.” The P3 effect was estimated by contrasting these two events. Please note that contrast with block 1—as used in other studies (Niedeggen et al., 2019)—is not possible because the event “ball reception of the nonintended player” is not defined.

The epochs were then baseline-corrected (−100 to 0 ms), and individual segments with ocular artifacts (EOGs > 50 µV) were automatically identified and excluded from the analysis (Niedeggen et al., 2019, 2023). Subsequently, in a second semiautomatic artifact rejection, trials with amplitudes exceeding ±80 µV of the active electrodes were flagged (Feng et al., 2018). Those marked trials due to high alpha activity or movement-related artifacts were excluded. In a final manual correction, trials with slow linear drifts affecting the baseline period or high-frequency bursts were inspected and excluded (Kawamoto et al., 2013; Weschke & Niedeggen, 2015). In a final manual correction, trials with slow linear drifts affecting the baseline period or high-frequency bursts were inspected and excluded. After rigorous artifact rejection, trials were averaged separately for the different ERP events.

For the event of “ball reception by the intended co-player,” the average number of trials analyzed was 19.11, with a mean rejection rate of 50.6% (CG: *M* = 20.92 trials, *SD* = 7.16, range 11–34 trials; EG1_disc_: *M* = 21.04 trials, *SD* = 7.39, range 10–36 trials; EG2_cont_: *M* = 15.36 trials, *SD* = 3.26, range 10–22 trials), while the event of “ball reception by the nonintended co-player” had a mean rejection rate of 36.3% with an average of 10.61 trials analyzed (CG: *M* = 12.58 trials, *SD* = 2.45, range 10–17 trials; EG1_disc_: *M* = 12.71 trials, *SD* = 2.42, range 10–17 trials; EG2_cont_: *M* = 6.56 trials, *SD* = 1.23, range 5–9 trials). Notably, based on previous studies (Kawamoto et al., 2013; Niedeggen et al., 2019), the number of ten single trials is sufficient to compute an averaged ERP signal with a sufficient signal/noise ratio to allow an analysis of the P3 effect. In the current study, this would have excluded the majority of participants in EG2_cont_. Therefore, to allow a comparison with the previous study on the preexposure effect (Fang et al., 2022), our data were analyzed in two steps: first, the preexposure effects were analyzed in the more stable CG and EG1_disc_. In a second analysis, the data from EG2_cont_ were additionally considered. Because of the low number of data points, the latter effects require a more cautious interpretation.

### Data analysis

All statistical analyses were performed by using SPSS (version 27, IBM) and Jamovi (version 0.9.4.2, Jamovi Development Team).

Self-reported data: Initially, a manipulation check was performed to assess whether the participants had noticed the changes in the frequency of the intervention. A 3 × 2 ANOVA was conducted, which comprised the between-participant factor of “group” with three levels (CG, EG1_disc_, and EG2_cont_) and the within-participant variable of “block” with two levels (block 1 and block 2). For ANOVA, the *p* values and degrees of freedom were adjusted according to the Greenhouse-Geisser correction. If a significant interaction was found, post hoc comparisons were conducted. Please note that these statistical procedures were also applied in the following steps of analysis.

The analysis of the changes in feelings (“control,” “negative mood,” “belonging,” and “personal power”) was then performed in two steps. Because the self-reports were already based on differential ratings (i.e., changes from block 1 to block 2, as mentioned above), we first conducted one-sample *t*-tests in CG to test whether self-reported threats induced by the transition to loss of control were consistent with previous findings (Niedeggen et al., 2019). Next, to test our hypotheses, each of the individual scales of interest was analyzed separately by using one-way ANOVAs with the between-factor “group” (CG, EG1_disc_, and EG2_cont_). A larger value of the mean difference score (Δ(Block 2 − Block 1)) reflects a larger increase in threat between the two successive blocks. A significant effect of the factor “group” allowed post hoc comparisons.

Given that the self-reported threats are closely linked to the negative mood in the previous preexposure study (Fang et al., 2022), Pearson’s correlation analyses between them also were conducted in this study.

To test whether the effects of preexposure depended on the type of the first threat (exclusion or loss of control), the actual data were compared with the preexposure effect observed in the previous study (Fang et al., 2022). The comparison included only the “negative mood” scale. Other scales were not considered, because the two studies focused on the impacts of different social threats on the processing of an upcoming new threat (loss of control ➔ exclusion vs. exclusion ➔ loss of control). Consequently, a 2 × 3 ANOVA was performed with two between-participant variables (“transition type” with two levels: intervention and exclusion, and “group” with three levels: CG, EG1_disc_, and EG2_cont_). A significant effect of the factor “group” and/or a significant interaction allowed post hoc comparisons.

E﻿RPs data: To identify temporal regions suitable for statistical analysis, we computed difference waves of grand-averaged ERPs in response to two distinct events in block 2: “ball reception by intended co-players” and “ball reception by nonintended co-players.” For the resulting difference in ERP waveforms (Δ(nonintended − intended)), the Global Field Power (GFP) index was determined, and a sustained activation was identified in the time range 220–450 ms, independent of group assignment. Within the sustained activation, two local maxima were identified by GFP (Fig. S1 in the *Supplementary Material*): The first peak in the GFP power (280 ms) is assumed to reflect the early P3, and the second peak at about 390 ms is referred to the late P3 in the following. To account for interindividual differences, we defined the first temporal window as an early P3 response ranging from 240 to 320 ms and the second temporal window as a late P3b-like response ranging from 350 to 430 ms. The length of the temporal window has been used in earlier Cyberball studies (Schuck et al., 2018; Weinbrecht et al., 2018).

For each time window, mean amplitudes were computed separately for the events “intended recipients” and “nonintended recipients” and electrodes. Following previous analyses of the P3 effect in Cyberball games (Niedeggen et al., 2019; Schuck et al., 2018), this analysis included the midline electrodes, Fz, Cz, and Pz. As stated, we initially performed 2 × 2 × 3 ANOVAs on the exported amplitude data of the ERP components. These analyses included the between-participant factor “group” (CG and EG1_disc_) and the within-participant factors “recipient” (intended and nonintended) and “electrode” (Fz, Cz, and Pz). Subsequently, in a second step, the analysis also included the EG2_cont_, resulting in a 3 × 2 × 3 analysis. A significant effect of the factor “group” and/or a significant interaction allowed post hoc comparisons.

Comparable to the scale “negative mood,” we also tested whether the ERP effects were differentially expressed for different preexposure threats. Therefore, we compared the current data (preexposure: exclusion) with the data of the previous study (preexposure: loss of control (Fang et al., 2022)). Please note that the P3 components are elicited by distinct target events (ball reception of participants vs. ball reception of co-players) in two separate studies and that these studies also vary in terms of the time windows used (previous study: 400–500 ms; current study: 350–430 ms) and the electrodes most responsive to activation (previous study: Cz, Pz, P7, P8; current study: Fz, Cz, Pz). These differences were accounted for by two steps in the analysis: first, in both studies we focused on a P3 effect, which means we computed a difference potential between two conditions. Second, we initially conducted z-score transformations on the P3 effects for each study. This procedure makes it possible to compare data from different studies by using a common scale. In this context, we applied z-score transformations to facilitate a meaningful analysis of the differences in P3 responses under varying preexposure conditions between the two studies. Subsequently, the statistical analysis also consisted of two steps. First, we performed a 2 × 2 ANOVA on the *z*-scores, excluding EG2_cont_ with “transition type” (intervention and exclusion) as one factor and “group” (CG and EG1_disc_) as the other. Second, a 2 × 3 ANOVA, including EG2_cont_, was conducted. A significant effect of the factor “group” and/or a significant interaction allowed post hoc comparisons.

## Results

**Fig. 2:**
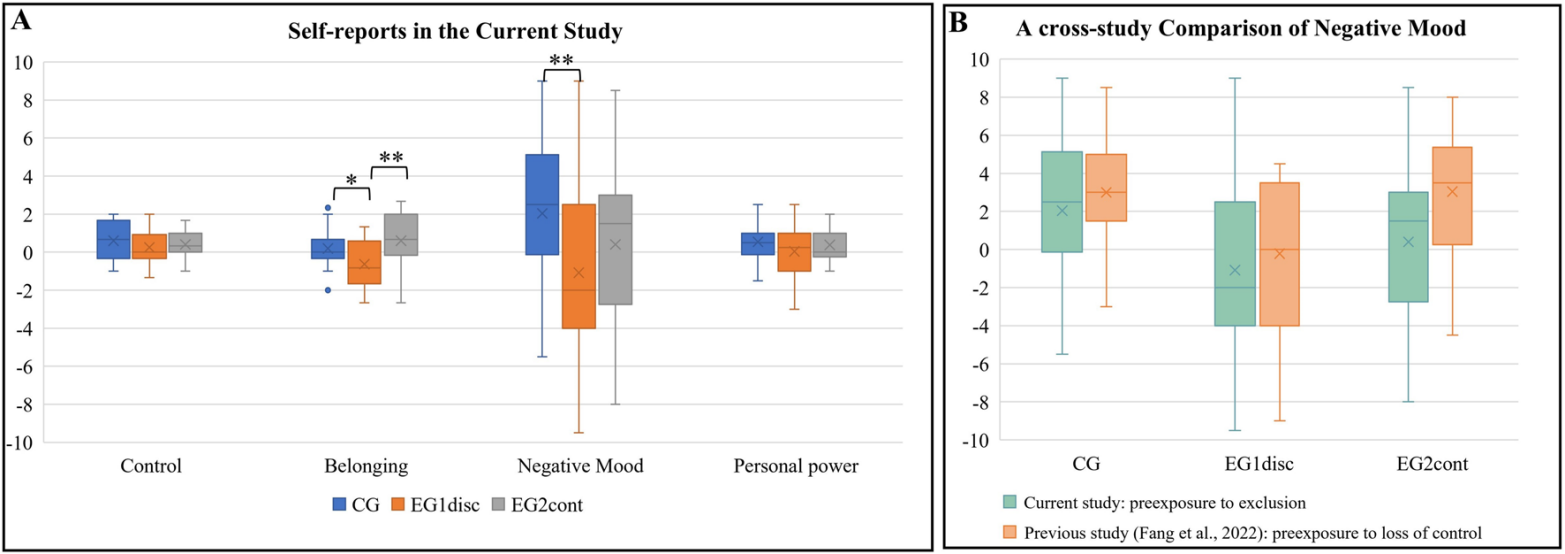
(**A**) Differences in retrospective self-reports (Δ(block2 − block1)) in the three groups. (**B**) Participants’ perceptions of “negative mood” in the current study (preexposure to exclusion) in relation to previous study (preexposure to loss of control; Fang et al., 2022). In all graphs, box-and-whiskers plots are used. *Notes.* CG = control group without preexposure; EG1_disc_ = experimental group 1 with discontinued preexposure; EG2_cont_ = experimental group 2 with continued first threat. *p < 0.05; **p < 0.01. Two data points were identified as outliers for “belonging” in (**A**) in the control group

To validate the effectiveness of the experimental manipulation and the specificity of the resulting threats to “control” and “belonging” as well as emotional arousal, across the three groups, we first conducted analyses of the retrospective self-reports (Table 1; Fig. 2).

**Table 1:** Descriptive statistics for self-reports and ERP data in three groups

			CG (*n* = 26)		EG1_disc_ (*n* = 24)		EG2_cont_ (*n* = 25)	
			*M (SE)*	*CI (95%)*	*M (SE)*	*CI (95%)*	*M (SE)*	*CI (95%)*
Estimated frequency (%)	LoC	B1	6.96 (2.47)	[2.05, 11.88]	8.33 (2.57)	[3.22, 13.45]	8.72 (2.51)	[3.71, 13.73]
		B2	30.35 (3.77)	[22.82, 37.87]	39.96 (3.93)	[32.13, 47.79]	30.24 (3.85)	[22.57, 37.91]
	BR	B1	34.96 (2.05)	[30.87, 39.06]	24.42 (2.14)	[20.16, 28.68]	23.12 (2.09)	[18.94, 27.30]
		B2	32.19 (2.16)	[27.90, 36.49]	31.75 (2.24)	[27.28, 36.22]	19.72 (2.20)	[15.34, 24.10]
NTQ: control			0.60 (0.19)	[0.20, 1.01]	0.25 (0.18)	[−0.13, 0.63]	0.40 (0.15)	[0.09, 0.71]
NTQ: belonging			0.19 (0.19)	[−0.21, 0.59]	−0.63 (0.26)	[−1.16, −0.09]	0.60 (0.30)	[−0.01, 1.21]
Negative mood			2.04 (0.72)	[0.55, 3.52]	−1.08 (0.85)	[−2.85, 0.68]	0.40 (0.84)	[−1.33 2.13]
Personal power			0.54 (0.21)	[0.12, 0.96]	0.04 (0.26)	[−0.49, 0.58]	0.38 (0.20)	[−0.03, 0.79]
Early P3 (µV)	Nonintended		6.19 (0.78)	[4.63, 7.75]	6.34 (0.82)	[4.72, 7.97]	5.13 (0.80)	[3.54, 6.72]
	intended		2.44 (0.48)	[1.48, 3.41]	3.63 (0.50)	[2.63, 4.63]	2.91 (0.49)	[1.93, 3.89]
Late P3 (µV)	Nonintended		6.49 (0.67)	[5.15, 7.84]	4.90 (0.70)	[3.50, 6.30]	4.86 (0.69)	[3.49, 6.23]
	intended		1.54 (0.48)	[0.59, 2.49]	1.91 (0.50)	[0.92, 2.90]	2.74 (0.49)	[1.77, 3.71]

The table presents descriptive statistics for self-reports separately for the three groups. The data are presented as differential scores, with the larger value of the mean difference score indicating higher levels of threat in block 2 compared to block 1. The table also includes ERP responses in the form of early P3 (240–320 ms) and late P3 (350–430 ms) expressions. *Notes*. CG: control group without preexposure; EG1_disc_: experimental group 1 with discontinued preexposure; EG2_cont_: experimental group 2 with continued first threat; LoC: loss of control; BP: ball reception; B1: Block 1; B2: Block 2; *M*: mean; *SE*: standard error; *CI*: confidence interval of the mean; NTQ: the need threat questionnaire

### Manipulation check

Participants in all three groups reported stronger feelings of loss of control in block 2 compared with block 1 (Table 1). A repeated-measures ANOVA revealed a significant main effect of “block,” *F*(1, 72) = 124.700, *p* < 0.001, *η*_p_^2^ = 0.634 (90% CI [0.517, 0.708]). The analysis revealed no significant main effect of “group” or interaction between “block” and “group”: *F*(2, 72) = 1.295, *p* = 0.280, *η*_p_^2^ = 0.035 (90% CI [0, 0.109]), and *F*(2, 72) = 1.806, *p* = 0.172, *η*_p_^2^ = 0.048 (90% CI [0, 0.131]), respectively. These results indicate that all participants reliably perceived an increase in the intervention from block 1 to block 2.

### Self-reports

#### Self-reports in the current study

At first, one-sample *t*-tests were applied exclusively for the CG to test whether the intervention elicited a selective threat to “control”—as reported in a previous study (Niedeggen et al., 2019). These results confirmed a significant increase on the scale “control” *t* (25) = 3.079, *p* = 0.005, *d* = 0.604 (95% CI [−0.19, 1.39]), but not on the scale “belonging” *t* (25) = 0.991, *p* = 0.331, *d* = 0.194 (95% CI [0.16, 1.80]).

To test our hypotheses, each scale of interest (“control,” “belonging,” “negative mood,” and “personal power”) was analyzed separately by using one-way ANOVAs with the between factor “group” (CG, EG1_disc_, EG2_cont_).

For the scale “control,” slight increases were obtained in all groups (Fig. 2A). However, the ANOVA did not reveal significant differences between groups, *F*(2, 72) = 1.000, *p* = 0.373, *η*_p_^2^ = 0.027 (90% CI [0, 0.095]).

For the scale “belonging,” the level of threat was reduced in EG1_disc_, but slightly enhanced in EG2_cont_ (Fig. 2A). A significant difference in the one-way ANOVA was found across the three groups, *F*(2, 72) = 5.961, *p* = 0.004, *η*_p_^2^ = 0.142 (90% CI [0.029, 0.253]). Further post hoc tests revealed that participants reported significantly less perceived threat to “belonging” in EG1_disc_ than in CG,* F*(1, 48) = 6.490, *p* = 0.014, *η*_p_^2^ = 0.119 (90% CI [0.014, 0.264]), and also than in EG2_cont_, *F*(1, 47) = 9.560, *p* = 0.003, *η*_p_^2^ = 0.169 (90% CI [0.036, 0.320]). However, no significant difference was found between CG and EG2_cont_, *F*(1, 49) = 1.340, *p* = 0.253, *η*_p_^2^ = 0.027 (90% CI [0.027, 0.134]).

“Negative mood” was increased with the transition from block 1 to block 2 in CG and EG2_cont_, but not in EG1_disc_ (Fig. 2A). The results of the one-way ANOVA revealed a significant difference across the three groups, *F*(2, 72) = 3.779, *p* = 0.028, *η*_p_^2^ = 0.095 (90% CI [0.006, 0.196]). Subsequent post hoc comparisons revealed that compared with CG, participants significantly reported less negative mood in EG1_disc_,* F*(1, 48) = 7.910, *p* = 0.007, *η*_p_^2^ = 0.142 (90% CI [0.023, 0.289]). However, no significant differences were found between CG and EG2_cont_, *F*(1, 49) = 2.200, *p* = 0.144, *η*_p_^2^ = 0.043 (90% CI [0, 0.162]), or between EG1_disc_ and EG2_cont_, *F*(1, 47) = 1.540, *p* = 0.221, *η*_p_^2^ = 0.032 (90% CI [0, 0.146]).

Finally, the perceptions of “personal power” were slightly increased in all groups (Fig. 2A), but the ANOVA did not indicate statistically significant differences between the three groups, *F*(2, 72) = 1.400, *p* = 0.254, *η*_p_^2^ = 0.038 (90% CI [0, 0.114]).

In addition, Pearson’s correlation analyses were used to check the correlations between the need for threats and negative mood. In terms of the threat to “control” and negative mood, there was a strong correlation between them in all three groups combined, *r* = 0.501, *p* < 0.001. However, for each group, significant correlations existed only in CG, *r* = 0.506, *p* = 0.008, and EG1_disc_, *r* = 0.657, *p* < 0.001, but not in EG2_cont_, *r* = 0.270, *p* = 0.191. In terms of the threat to “belonging” and negative mood, strong correlations were indicated between them in all three groups combined, *r* = 0.625, *p* < 0.001, and also in each group separately (CG: *r* = 0.610, *p* < 0.001, EG1_disc_: *r* = 0.594, *p* = 0.002, and EG2_cont_, *r* = 0.673, *p* < 0.001).

### Comparison of “negative mood” with previous preexposure study

The scale “negative mood” was used to compare the effect of preexposure to “loss of control” in the previous study (Fang et al., 2022) with the preexposure to “exclusion” employed in the current study. The data presented in Fig. 2B were analyzed by running a 2 (factor “transition type”: loss of control and exclusion) × 3 (factor “group”: CG, EG1_disc_ and EG2_cont_) ANOVA. The results showed that the main effect of “group” was highly significant, *F*(2, 139) = 9.075, *p <* 0.001, *η*_p_^2^ = 0.115 (90% CI [0.039, 0.194]), with clear increases in CG and EG2_cont_ as well as decreases in EG1_disc_. Results of post hoc tests showed that compared with CG, significantly less negative mood was reported in EG1_disc_, *F*(1, 94) = 17.188, *p <* 0.001, *η*_p_^2^ = 0.155 (90% CI [0.057, 0.263]), but not in EG2_cont_,* F*(1, 96) = 1.125, *p =* 0.292, *η*_p_^2^ = 0.012 (90% CI [0, 0.070]). In addition, participants in EG2_cont_ reported more negative mood than those in EG1_disc_, *F*(1, 94) = 8.045, *p =* 0.006, *η*_p_^2^ = 0.079 (90% CI [0.014, 0.175]).

The main effect of “transition type” also was significant, *F*(1, 139) = 5.626, *p* = 0.019, *η*_p_^2^ = 0.039 (90% CI [0.003, 0.103]) and indicated that the transition to exclusion elicited larger effects on negative mood.

However, the interaction term indicated that the type of preexposure (loss of control vs. exclusion) did not modulate these effects, *F*(2, 139) = 0.849, *p* = 0.430, *η*_p_^2^ = 0.012 (90% CI [0, 0.048]). In other words, negative mood was found to be enhanced by a transition to exclusion independently of the preexposure conditions (CG, EG1_disc_, and EG2_cont_).

### ERPs effects

#### Ball reception by intended and nonintended co-players in the current study

**Fig. 3:**
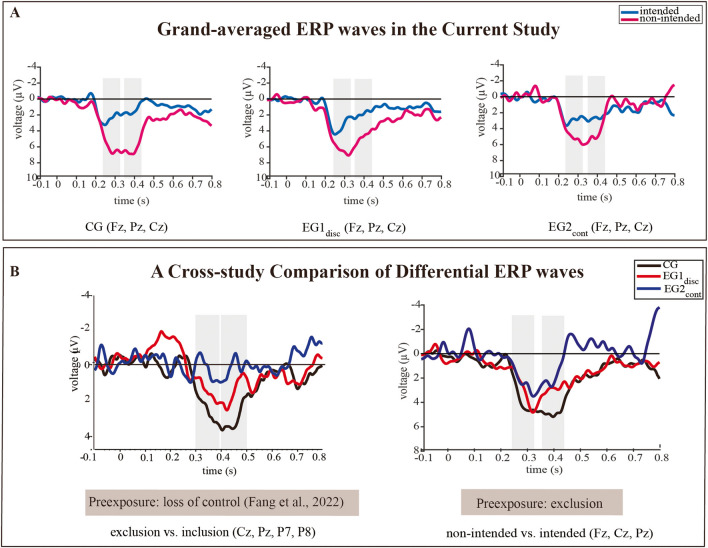
(**A**) Grand-averaged ERP effects elicited by the events “ball reception by intended co-player” and “ball reception by nonintended co-player” in block 2 at midline electrode leads (cluster: Fz, Cz, and Pz). (**B**) Differential ERP waves (Δ(nonintended − intended)) were calculated for three groups in the current study (preexposure: exclusion) and differential ERP waves (Δ(exclusion − inclusion)) were calculated for three groups in the previous study (preexposure: loss of control (Fang et al., 2022)). The time windows considered for analysis (this study: 240–320 ms and 350–430 ms; previous study: 300–400 ms and 400–500 ms) are highlighted. Details of the analysis and findings are provided in the Methods and Results sections. *Notes*. CG = control group without preexposure; EG1_disc_ = experimental group 1 with discontinued preexposure; EG2_cont_ = experimental group 2 with continued first threat

To explore the state of expected control, we examined the changes in the P3 responses in block 2 elicited by the experimental factor “recipient” (“ball reception by the intended co-player” vs. “ball reception by nonintended co-player”) (Niedeggen et al., 2019). Descriptive statistics for the ERP data are presented in Table 1, and the ERP effects in the three experimental groups are displayed in Fig. 3A. The results showed a temporally sustained positivity that extended from 220 to 450 ms following brief negativity at 180 ms. This positivity was consistently observed across the groups and was characterized by two distinct peaks in global field power over the time range analyzed, one in the early P3 time range (peak: 280 ms, time range for analysis: 240–320 ms) and the other in the late P3 segment (peak: 390 ms, time range for analysis: 350–430 ms), referring to the classical P3b.

### Early P3 Amplitude (240–320 ms)

A large P3 effect (Δ(nonintended - intended)), depicted in Fig. 3B (right side), in the early period, was observed in CG. The degree of the P3 effect gradually declined in EG1_disc_ and EG2_cont_. To consider the weaker signal-to-noise ratio in EG2_cont_ (reduced number of single trials, see Methods section), data were analyzed in two consecutive tests. The first ANOVA tested for differences between CG and EG1_disc_. The result showed a significant main effect of the “recipient,” i.e., the P3 amplitude was greater when the ball was received by nonintended co-players than intended co-players, *F*(1, 48) = 40.173, *p* < 0.001, *η*_p_^2^ = 0.456 (90% CI [0.275, 0.578]). However, we did not observe a significant main effect of the factor “group,” *F*(1, 48) = 0.817, *p* =0.370, *η*_p_^2^ = 0.017 (90% CI [0, 0.115]), or a significant interaction of the factors “group” and “recipient,”* F*(1, 48) = 1.027, *p* = 0.316, *η*_p_^2^ = 0.021 (90% CI [0, 0.124]).

In a second step, we also included the data of EG2_cont_. Again, the ANOVA indicated a significant main effect of “recipient” (nonintended > intended), *F*(1, 72) = 40.079, *p* < 0.001, *η*_p_^2^ = 0.358 (90% CI [0.211, 0.474]). Similarly, no significant interaction was found between the factors “group” and “recipient,”* F*(2, 72) = 0.994, *p* = 0.375, *η*_p_^2^ = 0.027 (90% CI [0, 0.095]), nor was there a significant main effect of the factor “group,” *F*(2, 72) = 0.838, *p* = 0.437, *η*_p_^2^ = 0.023 (90% CI [0, 0.087]).

Please note that the nonsignificant interaction and main effect of the factor “group” were not modulated by the location of the midline electrodes in the analysis of CG and EG1_disc_, “recipient” × “group” × “electrode” *F*(1.437, 68.956) = 0.696, *p* = 0.457, *η*_p_^2^ = 0.014 (90% CI [0, 0.080]) and “group” × “electrode” *F*(1.384, 66.440) = 0.339, *p* = 0.634, *η*_p_^2^ = 0.007 (90% CI [0, 0.062]), and the results were similar when the analysis included EG2_cont_, “recipient” × “group” × “electrode” *F*(2.885, 103.867) = 0.353, *p* = 0.779, *η*_p_^2^ = 0.010 (90% CI [0, 0.033]) and “group” × “electrode” *F*(2.751, 99.050) = 0.238, *p* = 0.854, *η*_p_^2^ = 0.007 (90% CI [0, 0.024]).

### Late P3 Amplitude (350–430 ms)

The late P3 effect, contrasting the events “intended” and “nonintended” recipients, was clearly expressed in CG but appears to be reduced in EG1_disc_ and—even more—diminished in EG2_cont_ (Fig. 3B, right side). The first step of analysis included CG and EG1_disc_ and revealed a significant main effect of the factor “recipient” (nonintended > intended), *F*(1, 48) = 77.081, *p* < 0.001, *η*_p_^2^ = 0.616 (90% CI [0.461, 0.706]) and also a significant interaction between the factors “group” and “recipient,” *F*(1, 48) = 4.683, *p* = 0.035, *η*_p_^2^ = 0.089 (90% CI [0.003, 0.228]), but no significant main effect of “group,” *F*(1, 48) = 0.756, *p* = 0.389, *η*_p_^2^ = 0.016 (90% CI [0, 0.112]). The post hoc analyses for the interaction effect showed that the P3 effect (Δ(nonintended − intended)) was significantly reduced in EG1_disc_ compared with CG, *F*(1, 48) = 4.683, *p* = 0.035, *η*_p_^2^ = 0.089 (90% CI [0.003, 0.228]).

In the second step, ANOVA also included EG2_cont_. Again, the significant main effect of the factor “recipient” (nonintended > intended), *F*(1, 72) = 76.222, *p* < 0.001, *η*_p_^2^ = 0.514 (90% CI [0.376, 0.609]), as well as its interaction with the factor “recipient” x “group,” *F*(2, 72) = 4.868, *p* = 0.010, *η*_p_^2^ = 0.119 (90% CI [0.017, 0.226]), were confirmed. Still no significant main effect of “group” was observed, *F*(2, 72) = 0.389, *p* = 0.679, *η*_p_^2^ = 0.011 (90% CI [0, 0.057]). The further post hoc analyses showed that the P3 effect (Δ(nonintended − intended)) was significantly reduced in both experimental groups compared with the control group: CG vs. EG1_disc_, *F*(1, 48) = 4.683, *p* = 0.035, *η*_p_^2^ = 0.089 (90% CI [0.003, 0.228]); CG vs. EG2_cont_, *F*(1, 49) = 9.450, *p* = 0.003, *η*_p_^2^ = 0.162 (90% CI [0.034, 0.310]). But no significant difference in the P3 effect was found between EG1_disc_ and EG2_cont,_ *F*(1, 47) = 0.773, *p* = 0.384, *η*_p_^2^ = 0.016 (90% CI [0, 0.115]).

In addition, the analyses revealed that the critical interaction between the factors “recipient” and “group” was not modulated by the location of the midline electrodes, as indicated by the nonsignificant results in both the analysis of CG and EG1_disc_, *F*(1.513, 72.606) = 0.028, *p* = 0.941, *η*_p_^2^ = 0.001 (90% CI [0, 0.004]), and the analysis of all three groups, *F*(2.764, 99.487) = 0.101, *p* = 0.951, *η*_p_^2^ = 0.003 (90% CI [0, 0.004]).

### Comparison of the differences in ERP waves between the previous study and the current study

To test whether the preexposure conditions “loss of control” and “exclusion” modulate the processing of upcoming threats, we compared the current ERP results with the results of the previous study (Fang et al., 2022). The corresponding ANOVA was conducted to compare the P3 effects between “intended vs. nonintended” events observed in the current study with those between “inclusion vs. exclusion” events observed in a previous study (Fang et al., 2022) (Fig. 3B, left-side). As mentioned, due to differences in the time windows showing the most significant P3 effect, *z*-score transformations of the difference waves were required for each study.

The first analysis only considered the groups CG and EG1_disc_. The results showed a significant main effect of “group” in which the P3 effects (differential waves) were weaker in EG1_disc_ than in CG, *F*(1, 92) = 8.816, *p =* 0.004, *η*_p_^2^ = 0.087 (90% CI [0.017, 0.187]), but no significant main effect of “transition type,” *F*(1, 92) = 0.084, *p* = 0.772, *η*_p_^2^ = 0.001 (90% CI [0, 0.032]), or interaction effect, *F*(1, 92) = 0.049, *p* = 0.825, *η*_p_^2^ = 0.001 (90% CI [0, 0.026]).

The second step of analysis considered EG2_cont_. The ANOVA also indicated a significant main effect of “group,” *F*(2, 139) = 9.931, *p <* 0.001, *η*_p_^2^ = 0.125 (90% CI [0.045, 0.205]). Post hoc analyses of the main effect of “group” revealed that the P3 effects were greater in groups CG than EG1_disc_, *F*(1, 94) = 9.030, *p =* 0.003*, η*_p_^2^ = 0.088 (90% CI [0.018, 0.186]), as well as EG2_cont_, *F*(1, 96) = 17.900, *p <* 0.001,* η*_p_^2^ = 0.157 (90% CI [0.060, 0.265]), whereas the groups EG1_disc_ and EG2_cont_ did not show significant differences, *F*(1, 94) = 2.700, *p =* 0.104,* η*_p_^2^ = 0.028 (90% CI [0, 0.102]). Importantly, the comparison of studies did not provide evidence that the processing of a specific threat (exclusion or loss-of-control) was specifically modulated by different preexposure (interaction “group” x “transition type,” *F*(2, 139) = 0.076, *p* = 0.927, *η*_p_^2^ = 0.001 (90% CI [0, 0.006])). Due to the *z*-normalization, ERP amplitudes were comparable for both “transition types,” *F*(1, 139) = 0.007, *p* = 0.931, *η*_p_^2^ = 0.000 (90% CI [0, 0.004]).

## Discussion

The purpose of this study was to explore the impact of previous social exclusion on the processing of subsequent loss-of-control events. The results supported our first hypothesis, indicating that the P3 effect (Δ(nonintended recipient – intended recipient)) observed in the control group (CG) in response to the transition to loss of control was attenuated in the experimental groups due to the presence of the preexposure threat. Contrary to our expectations, this reduction in the P3 effect did not correspond to a significant decrease in the expected self-reported threat to “control” but to a noticeable decrease in “negative mood.” Furthermore, the continuation (EG2_cont_) or discontinuation (EG1_disc_) of the preexposure to social exclusion did not significantly influence either the P3 effect or the self-reports in the two experimental groups, contradicting our second hypothesis.

Moreover, an additional exploratory analysis comparing the expression of the P3 effects between this study and the previous study (Fang et al., 2022) confirmed that both preexposure conditions (exclusion and loss of control) influenced on the P3 effects triggered by the subsequent threat. In both preexposure studies, the reduction in the P3 effect induced by preexposure did not predict the decrease in the corresponding need threat scale. Instead, the expression of the specific need threat observed in the self-reports was closely related to the arousal of negative mood. In terms of expressing negative mood, preexposure to exclusion had a stronger influence than preexposure to loss of control.

The implications of these findings will be elaborated on in the following sections.

### ERP-effects of preexposure on the processing of loss of control

First, a clear P3 effect was observed in the “intervention” setting defined by loss of control. These results in CG replicated those findings of a previous intervention Cyberball study (Niedeggen et al., 2019), suggesting that P3 also signals interferences in social exchange. Comparable to the processing of a target event in the exclusionary Cyberball (Weschke & Niedeggen, 2015), the expression of the P3 effect can be related to the violation of the subjective expectation of control in social interaction (Niedeggen et al., 2019).

Compared with CG, P3 effects induced by the intervention attenuated in both experimental groups due to experiencing a preexposure to an unrelated threat (EG1_disc_ and EG2_cont_). Although all groups, in the present study, experienced loss of control in the second block, the differential expression of the P3 effect can be attributed to the preceding experience of social exclusion, which may have lowered individuals’ expectations of participation (Niedeggen et al., 2017; Weschke & Niedeggen, 2015) and prepared themselves for the subsequent loss of control. In other words, the P3 effect serves as an indicator of participants’ expectancy state (Niedeggen et al., 2023; Schuck et al., 2018), which can be modulated by the prior experience of another negative social event (Fang et al., 2022).

This finding aligns with preliminary evidence from the previous preexposure study (Fang et al., 2022). Importantly, a statistical analysis embracing the data of both preexposure studies revealed a reliable preexposure effect, as manifested in the attenuated P3 effects. These preexposure effects support the inconsistency compensation approach (Proulx et al., 2012), which posits that any given inconsistency (in this case, two different types of preexposure) can elicit a common pattern of aversive arousal. This arousal usually drives a palliative response to violated expectations. These assumptions are specified in a recently proposed expectancy violation (ViolEx) model of expectation maintenance and updating (Panitz et al., 2021). The model proposes that individuals can cope with expectation violations through accommodation processes that involve updating their expectations to minimize discrepancies between internal expectations and encountered situational outcomes. The combination of both preexposure studies indicates that a preexposure threat can foster adaptive accommodation processes in response to expectancy violations independently of the nature of the threat involved. As a result, the magnitude of expectancy violation is diminished in subsequent novel threats.

However, not all hypotheses were supported by the current data. Based on our previous findings (Fang et al., 2022), we hypothesized that the modulation of the P3 effect would depend on whether the preexposure was discontinued or continued. According to the ViolEx model (Panitz et al., 2021), discontinuation of the preexposure may restore expectancy and initiate a new adaptation process, whereas continuation of the preexposure may reinforce initial internal anticipatory reactions. Contrary to this assumption, the continuation or discontinuation of the preexposure (EG1_cont_ vs. EG2_disc_) did not significantly modulate the P3 effects elicited by the loss-of-control threat.

Nevertheless, the current data probably do not allow us to refuse the idea of an “offset” effect of the preexposure threat. Most of all, two methodological distinctions between the two preexposure studies must be considered. First, the previous study (Fang et al., 2022) examined the P3 effect by comparing one event (ball reception by participants) across blocks, whereas the actual study compared two events (intended vs. nonintended recipient) within one block. Previous research found that block-based cues (between-block design) promoted sustained preparatory effort for target stimuli, whereas trial-based cues (within-block design) elicited not only sustained but also transient preparatory effort (Kostandyan et al., 2019). Therefore, in these two preexposure studies, the adaptation of internal anticipatory reactions within blocks versus across blocks may affect the expression of the P3 effect. Second, the difference in recognizing different social threats (exclusion and loss of control) could affect the pace of adaptation of internal anticipatory reactions. Specifically, the intervention overriding participants’ decision should be recognized immediately after it occurs, whereas previous research reveals that participants’ mood begins to drop sharply after approximately 20 s (approximately 6 throws) of not receiving the ball (Williams, 2009; Williams & Jarvis, 2006). This could imply that the preexposure impact of social exclusion may be relatively slow to register, potentially explaining its limited moderation of P3 effects in both EG1_disc_ and EG2_cont_ in the current study.

Taken together, the preexposure threats may decrease individuals’ expectancy violations in the face of a new social threat, as evidenced by the reduced P3 effects. Although the expected effect of preexposure status (continuation vs. discontinuation) on the P3 effect was not supported in this study, we cannot completely rule out the possibility that a temporal overlap of the threats could affect the anticipatory reactions in a common expectancy system. Moreover, the preexposure effect provides further evidence for the interaction of different threats in a common expectancy system. In contrast to the decrease in the P3 effect due to the *subsequent* processing of two different social threats reported in the present study, a recent Cyberball study requesting the *simultaneous* processing of two social threats (Niedeggen et al., 2023) reported a significant increase in the P3 effect. In other words, whereas preexposure induces an adaptation of the expected social participation and control, the concurrent onset of two threats results in a sensitization effect.

### Effects of preexposure in self-reports

The self-reports in this study replicated earlier findings (Niedeggen et al., 2019) regarding the effect induced by the intervention in the modified Cyberball setup. The intervention in the participant’s choice of the recipient of the ball throw selectively increased the threat to the need for “control” but not “belonging.” However, in a preexposure condition, a reduction of the P3 effect was not associated with a corresponding reduction in the self-reported threat to “control” (see Section 3 in the *Supplementary Material*). Instead, the modulation was expressed in the self-reported levels of “negative mood” that were strongly associated with the threat to “control,” which were significantly elevated in the control group but not significantly increased in both preexposure groups.

This pattern of results might indicate the lack of specificity and sensitivity of the subscale “control” in a combined exclusion and intervention Cyberball paradigm. The lack of specificity may be due to the fact that the preexposure to exclusion affects not only the subscale “belonging” but also spreads to other subscales, such as “control” (Niedeggen et al., 2019; Williams, 2007). The lack of sensitivity, in contrast to the ERPs, might be attributed to the temporal delay in evaluating the preceding interaction. Specifically, in contrast to the online monitoring of brain activity, the post hoc evaluation of the personal state might be more susceptible to social desirability bias (Latkin et al., 2017).

While the “control” subscale measures a single valence related to the basic need (Williams et al., 2000), the “negative mood” scale assesses a broader construct that encompasses a general dimension of subjective distress and unpleasant engagement, including anxiety, disgust, sadness, dissatisfaction, etc. (Solomon & Stone, 2002; Watson et al., 1988; Williams et al., 2000). These properties might contribute to the increased sensitivity of the scale “negative mood” and lead to strong associations with the need threat to “control,” even with the need threat to “belonging.” In other words, although responses to self-reported threats cannot be predicted reliably based on ERP effects in the preexposure situation, our data suggest that the experience of social exclusion diminishes the negative affective response to a subsequent loss-of-control threat, which also may be related to changes in expectancy levels.

A joint analysis of the effect of preexposure threat on “negative mood”—also including the results of the preceding preexposure study (Fang et al., 2022)—provides a better understanding of the general process. Both studies consistently demonstrated that the preexposure threat dampens the effect of the succeeding social threat on negative emotions. According to the ViolEx model (Panitz et al., 2021), expectation violations can be quantified by their magnitude and direction (Schultz et al., 1997; Yacubian et al., 2006). When the offset of the preexposure threat coincides with the onset of a new threat, the regaining of “control” or “belonging” can be defined as a pleasant surprise that moderates the negative expectancy violation elicited by the new threat, thereby reducing the a posteriori evaluation of negative mood. However, if the preexposure threat persists and overlaps with the new threat, the preparatory effect is validated, but the double-threat scenario is worse than expected, resulting in an additional degree of negative mood.

Furthermore, the joint analysis also indicated differences in the valence of the two threats; self-reported negative mood was expressed more strongly if participants experienced a transition to exclusion as compared to a transition to loss of control. A greater impact of exclusion as contrasted to loss-of-control on negative mood also was observed in a recent Cyberball study focusing on the processing of concurrent threats (Niedeggen et al., 2023). This suggests that the two types of threats may underlie differences in the severity of “social injury” (Bernstein & Claypool, 2012); social exclusion may be regarded as a highly severe social injury and elicit more emotional pain (Blackhart et al., 2009).

## Limitations

The following limitations must be considered when interpreting the results. First, as pointed out, because of the experimental design, the number of trials available for ERP analysis in EG2_cont_ is restricted, and experimental results considering this group must be treated more cautiously. To minimize its impacts, we considered this factor in the two-stepped analysis. Second, in addition to the P3 component, other ERP components associated with the conflicting stimulus have not been addressed. For example, attentional processes (N2, Hudac, 2019) and anticipatory responses (Contingent Negative Variation (CNV); van den Berg et al., 2014) might be expected. However, these components were not included in this study due to their limited prominence in the previous studies (Fang et al., 2022; Niedeggen et al., 2019, 2023), and thus hypotheses regarding them were not formulated. Third, the NTQ scale used in this study lacks specificity when examining threats to “control” (as discussed earlier), as both exclusion and loss of control can affect the need for “control” (Niedeggen et al., 2019; Weschke & Niedeggen, 2016; Williams et al., 2000). Fourth, given that Cyberball involves only virtual players, it does not reflect the complexity of real-life social interactions. Thus, increasing the naturalness of the setting in future research may improve ecological validity in this field. Fifth, we acknowledge as a limitation of this study that we cannot account for individual differences in personality traits, such as social threat sensitivity and anxiety. Considering this factor will require a much larger sample of participants and would be an interesting avenue for future research. In addition, as exemplified by the study conducted by Simard and Dandeneau (2018), it is a promising approach to consider personality traits as covariates in future Cyberball experiments. Furthermore, although participants were recruited from various native-speaking countries, they were all undergraduate students, and their responses to social threats may have been influenced by their educational level. To enhance the generalizability of the findings, larger and more diverse samples are needed in future research.

## Conclusions

The present study enriches the existing evidence that preexposure to one social threat influences the processing of a subsequent different social threat. Such preexposure to a social threat has the potential to induce an adaption of expectations related to social participation and control, thereby reducing the element of surprise associated with an upcoming novel threat. Consequently, both electrophysiological and behavioral responses tend to be less expressed in the context of preexposure. These results support the notion that preexposure triggers a mechanism designed to compensate for inconsistencies and palliating expectation violations (Proulx et al., 2012) and also can be integrated into the theoretical framework of the ViolEx model (Panitz et al., 2021). The impact of social threats on mental health issues has already been discussed, and a sustained negative-expectancy bias has been linked to clinical states (Cao et al., 2015). However, further longitudinal studies are necessary to decide whether preexposure-induced adaptation also plays a role in modulating the impact of social threats on mental health issues.

### Supplementary Information

Below is the link to the electronic supplementary material.

Supplementary file1 (DOCX 109 KB)

## Data Availability

Preprocessed EEG data, self-reports, as well as questionnaires are available at https://osf.io/dbmcr/.

## Code availability

The source code of the experimental procedure is available at https://osf.io/dbmcr/.
