# Supplementary material for: Preexposure to one social threat alters responses to another social threat: Behavioral and electrophysiological evidence
Source: Cogn Affect Behav Neurosci. 2024 Jan 10;24(1):126–42. doi: 10.3758/s13415-023-01151-y (PMC10827860; doi:10.3758/s13415-023-01151-y)
Supplement: Supplementary file 1 — Supplementary file1 (DOCX 109 KB) [file 13415_2023_1151_MOESM1_ESM.docx]

***Supplementary Material***

**Preexposure to one social threat alters responses to another social threat: behavioral and electrophysiological evidence**

**Xu Fang^*^, Rudolf Kerschreiter, Yu-Fang Yang, Michael Niedeggen**

*** Correspondence:** Xu Fang: [xu.fang@fu-berlin.de](mailto:xu.fang@fu-berlin.de)

# **Section 1:** **Distribution of participants**

All participants with different mother tongue were randomly assigned and equally distributed to the three group. The distribution in different groups is as follows:

**Table S1** Distribution of participants. *Notes*. CG: control group without preexposure; EG1_disc_: experimental group 1 with discontinued preexposure; EG2_cont_: experimental group 2 with continued first threat.

|  | German | English | Chinese | total |
| --- | --- | --- | --- | --- |
| CG | 19 | 5 | 2 | 26 |
| EG1_disc_ | 13 | 6 | 5 | 24 |
| EG2_cont_ | 10 | 9 | 6 | 25 |
| total | 42 | 20 | 13 | 75 |

# **Section 2: Global Field Power (GFP) plots**

The separation of the P3 component into early and late parts was determined by the Global Field Power (GFP) index. The analysis was based on the grand-averaged ERPs in block 2 (Δ (non-intended – intended)) – combining all three groups. In the figures below, two distinct local maxima were identified within the time range of 220–450 ms. The first local maximum occurred at 280 ms, followed by a second peak at 390 ms.

**
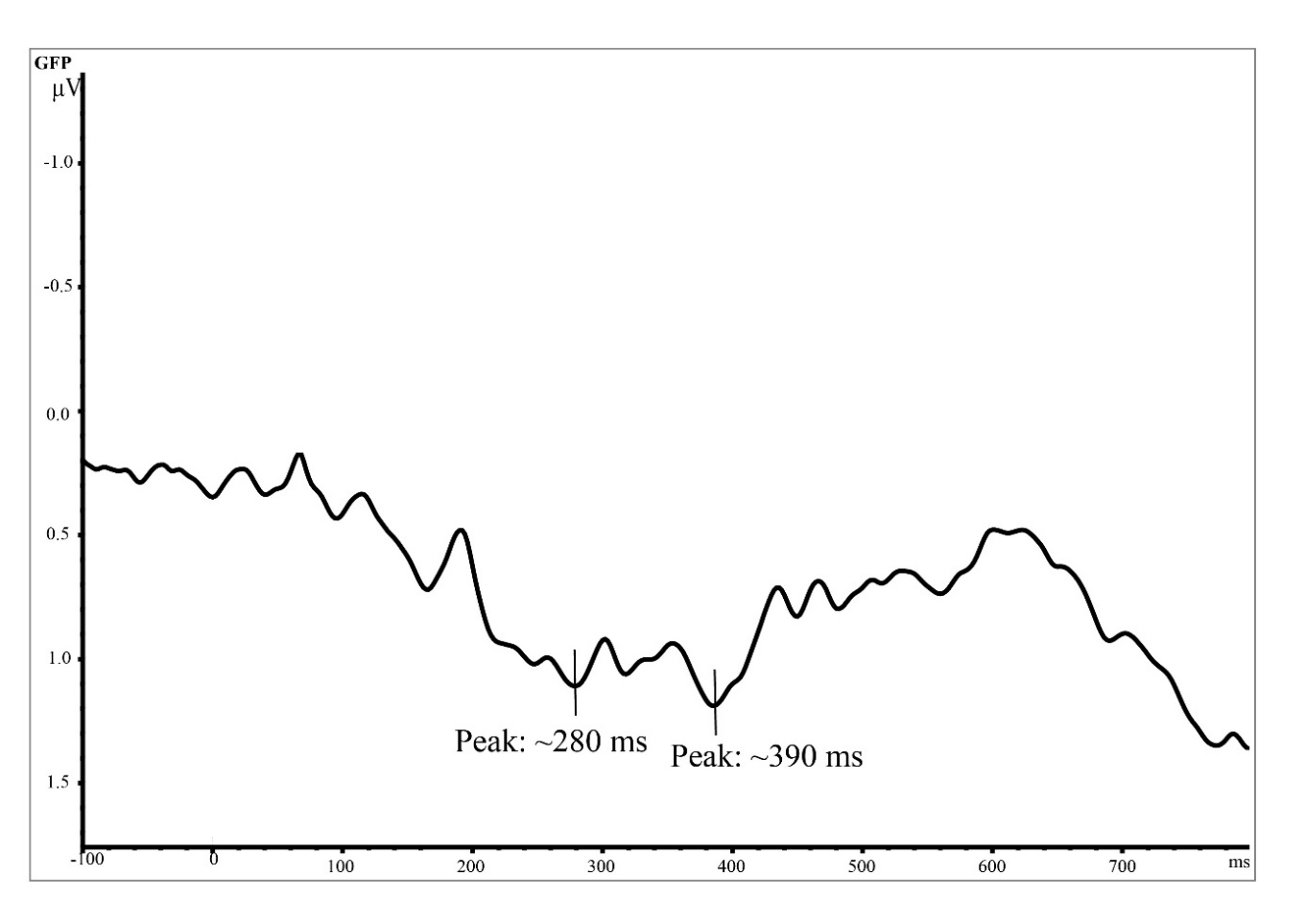
**

**Fig. S1** Average GFP of difference waves (Δ (non-intended – intended)) among three groups. Two distinct local maxima were identified within the time range of 220–450 ms. The first local maximum occurred at 280 ms, followed by a second peak at 390 ms.

# **Section 3: Corrections between P3 effects and self-reports**

We didn’t observe a similar pattern between the self-reported threat to “control” and P3 effects with the transition to intervention in the three groups. Pearson’s correlation analysis also didn’t indicate significant correlations between them, for the early P3 and “control” in three groups, *r* = −0.049, *p* = 0.678, and in each group, CG, *r* = −0.010, *p* = 0.962; EG1_disc_, *r* = −0.021, *p* = 0.923; EG2_cont_, *r* = −0.188, *p* = 0.367; for the late P3 and “control” in three groups, *r* = 0.029, *p* = 0.804, and in each group, CG, *r* = −0.083, *p* = 0.686; EG1_disc_, *r* = −0.105, *p* = 0.626; EG2_cont_, *r* = 0.167, *p* = 0.424.

Although there was a similar pattern in the “negative mood” and P3 effects with the transition to intervention in the three groups, Pearson’s correlation analysis didn’t indicate strong correlations between them, for the early P3 and “negative mood” in three groups, *r* = −0.029, *p* = 0.804, and in each group, CG, *r* = −0.043, *p* = 0.834; EG1_disc_, *r* = −0.045, *p* = 0.836; EG2_cont_, *r* = −0.159, *p* = 0.447; for the late P3 and “negative mood” in three groups, *r* = −0.017, *p* = 0.886, and in each group, CG, *r* = −0.048, *p* = 0.816; EG1_disc_, *r* = −0.116, *p* = 0.591; EG2_cont_, *r* = −0.102, *p* = 0.627.

# **Section 4: Bayesian analysis for P3 effects and self-reports**

The Bayesian analysis was conducted using the jsp tool in Jamovi (version 0.9.4.2, Jamovi Development Team). We performed three-level and two-level Bayesian ANOVAs on self-reported difference scores (Δ (block 2 – block 1)) and P3 difference waves (Δ (non-intended – intended)), respectively. The Bayes factors (*BF*_10_) are presented in the table below. To facilitate a comparison of the results of our frequentist ANOVA, we also provide the corresponding *F*-values in the table. Asterisks indicate significance: * *p* < .05; ** *p* < .01. As can be seen from the table, Bayesian analysis complemented the frequentist approach, revealing a similar pattern of preexposure effects in both methods.

**Table S2** Statistical results of Bayesian ANOVAs and frequentist ANOVA on self-reported difference scores (Δ (block 2 – block 1)) and P3 difference waves (Δ (non-intended - intended)). *F*-values are for frequentist ANOVA and BF_10_-values are for Bayesian ANOVAs. *Notes*. CG: control group without preexposure; EG1_disc_: experimental group 1 with discontinued preexposure; EG2_cont_: experimental group 2 with continued first threat. * *p* < .05; ** *p* < .01.

|  | |  | **ANOVA for three group** | |  | **ANOVA for two groups** | | | | | | | |
| --- | --- | --- | --- | --- | --- | --- | --- | --- | --- | --- | --- | --- | --- |
|  | |  | **CG vs. EG1_disc_ vs. EG2_cont_** | |  | **CG vs. EG1_disc_** | |  | **CG vs. EG2_cont_** | |  | **EG1_disc_ vs. EG2_cont_** | |
|  | |  | ***F*** | ***BF_10_*** |  | ***F*** | ***BF_10_*** |  | ***F*** | ***BF_10_*** |  | ***F*** | ***BF_10_*** |
| NTQ: control | |  | 1.000 | 0.246 |  | 1.72 | 0.569 |  | 0.674 | 0.370 |  | 0.410 | 0.337 |
| NTQ: belonging | |  | 5.961** | 9.930 |  | 6.490* | 3.700 |  | 1.340 | 0.485 |  | 9.560** | 11.61 |
| Negative mood | |  | 3.779* | 2.020 |  | 7.910** | 6.340 |  | 2.200 | 0.688 |  | 1.530 | 0.533 |
| Personal power | |  | 1.400 | 0.310 |  | 2.300 | 0.719 |  | 0.307 | 0.318 |  | 1.090 | 0.444 |
| Early P3 (μV) | Δ (non-intended – intended) |  | 0.994 | 0.246 |  | 1.030 | 0.431 |  | 1.810 | 0.588 |  | 0.172 | 0.306 |
| Late P3 (μV) | Δ (non-intended – intended) |  | 4.868* | 4.590 |  | 4.680* | 1.850 |  | 9.450** | 11.270 |  | 0.773 | 0.391 |
